# BIM and mTOR expression levels predict outcome to erlotinib in EGFR-mutant non-small-cell lung cancer

**DOI:** 10.1038/srep17499

**Published:** 2015-12-07

**Authors:** Niki Karachaliou, Jordi Codony-Servat, Cristina Teixidó, Sara Pilotto, Ana Drozdowskyj, Carles Codony-Servat, Ana Giménez-Capitán, Miguel Angel Molina-Vila, Jordi Bertrán-Alamillo, Radj Gervais, Bartomeu Massuti, Teresa Morán, Margarita Majem, Enriqueta Felip, Enric Carcereny, Rosario García-Campelo, Santiago Viteri, María González-Cao, Daniela Morales-Espinosa, Alberto Verlicchi, Elisabetta Crisetti, Imane Chaib, Mariacarmela Santarpia, José Luis Ramírez, Joaquim Bosch-Barrera, Andrés Felipe Cardona, Filippo de Marinis, Guillermo López-Vivanco, José Miguel Sánchez, Alain Vergnenegre, José Javier Sánchez Hernández, Isabella Sperduti, Emilio Bria, Rafael Rosell

**Affiliations:** 1 Instituto Oncológico Dr Rosell, Quiron-Dexeus University Hospital, Barcelona, Spain; 2 Pangaea Biotech, Barcelona, Spain; 3 Department of Medical Oncology, University of Verona, Azienda Ospedaliera Universitaria Integrata, Verona, Italy; 4 Pivotal, Madrid, Spain; 5 Centre François Baclesse, Caen, France; 6 Hospital General de Alicante, Alicante, Spain; 7 Catalan Institute of Oncology, Hospital Germans Trias i Pujol, Badalona, Spain; 8 Hospital de Sant Pau, Barcelona, Spain; 9 Hospital Vall d’Hebron, Barcelona, Spain; 10 Complexo Hospitalario Universitario La Coruña, Spain; 11 Ospedale Santa Maria delle Croci, Ravenna, Italy; 12 Department of Medical and Surgical Sciences, Institute of Respiratory Diseases, University of Foggia, Italy; 13 Medical Oncology Unit, Human Pathology Department, University of Messina, Italy; 14 Catalan Institute of Oncology, Hospital Josep Trueta, Girona, Spain; 15 Clinical and Traslational Oncology Group, Institute of Oncology, Clínica del Country, Bogotá, Colombia; 16 Direttore, Divisione di Oncologica Toracica, Istituto Europeo di Oncologia—IEO, Milano, Italy; 17 Chief, Medical Oncology Service, Hospital de Cruces, Barakaldo, Vizcaya, Spain; 18 Medical Oncology Service, Hospital de la Princesa, Madrid, Spain; 19 Hôpital du Cluzeau, Limoges, France; 20 Unidad de Investigación en Salud Pública CIDICS-UANL; 21 Biostatistics, Regina Elena National Cancer Institute, Rome; 22 Molecular Oncology Research (MORe) Foundation, Barcelona, Spain; 23 Germans Trias i Pujol Health Sciences Institute and Hospital, Campus Can Ruti.

## Abstract

BIM is a proapoptotic protein that initiates apoptosis triggered by EGFR tyrosine kinase inhibitors (TKI). mTOR negatively regulates apoptosis and may influence response to EGFR TKI. We examined mRNA expression of *BIM* and *MTOR* in 57 patients with *EGFR*-mutant NSCLC from the EURTAC trial. Risk of mortality and disease progression was lower in patients with high *BIM* compared with low/intermediate *BIM* mRNA levels. Analysis of *MTOR* further divided patients with high *BIM* expression into two groups, with those having both high *BIM* and *MTOR* experiencing shorter overall and progression-free survival to erlotinib. Validation of our results was performed in an independent cohort of 19 patients with *EGFR*-mutant NSCLC treated with EGFR TKIs. In *EGFR*-mutant lung adenocarcinoma cell lines with high BIM expression, concomitant high mTOR expression increased IC_50_ of gefitinib for cell proliferation. We next sought to analyse the signalling pattern in cell lines with strong activation of mTOR and its substrate P-S6. We showed that mTOR and phosphodiesterase 4D (PDE4D) strongly correlate in resistant *EGFR*-mutant cancer cell lines. These data suggest that the combination of EGFR TKI with mTOR or PDE4 inhibitors could be adequate therapy for *EGFR*-mutant NSCLC patients with high pretreatment levels of BIM and mTOR.

Since the introduction of erlotinib and gefitinib into clinical practice, metastatic epidermal growth factor receptor (*EGFR)* positive lung cancer patients can be offered therapeutic alternatives with proven superiority over platinum-based chemotherapy^1^,^2^. The EURTAC trial demonstrated efficacy of erlotinib over chemotherapy for first-line treatment of European advanced *EGFR*-mutant non-small-cell lung cancer (NSCLC) patients^1^. However, many patients have no response at all and response is short-lived for most of those who do. The emergence of the T790M *EGFR* gatekeeper mutation or activation of bypass signalling pathways have been identified as the main mechanisms of resistance to EGFR tyrosine kinase inhibitors (TKI)^3^,^4^.

It is recognized that TKIs eliminate tumour cells by inducing a form of cell death called apoptosis, which is governed by the B-cell lymphoma protein 2 (Bcl-2) family of proteins and mitochondria^5^. The Bcl-2 family is composed of two types of proteins; anti-apoptotic members like Bcl-2, Bcl-xL and Mcl-1 and pro-apoptotic members divided into effectors and BH3-only proteins. The Bcl-2 interacting mediator of cell death (BIM) is a BH3-only protein that directly activates the ultimate effectors of apoptosis BAK (BCL-2 antagonist or killer) and BAX (BCL-2-associated X protein)^6^. *EGFR* mutations activate mitogen-activated protein kinase (MAPK)/ extracellular signal–regulated kinase 1/2 (ERK1/2) and phosphatidylinositol 3′ -kinase-AKT (PI3K/AKT) pro-survival pathways. BIM, a well-known target of MAPK signalling, is a mediator of tumour cell death in response to targeted therapies^7^ (Fig. 1). Faber *et al.*, were the first to demonstrate that patients with *EGFR*-mutant NSCLC and low *BIM* expression derive less clinical benefit from EGFR inhibitors^5^. We identified high levels of *BIM* mRNA expression as a predictive marker of response, progression-free survival (PFS) and overall survival (OS) in erlotinib-treated *EGFR*-mutant NSCLC patients^8^.

RAF or MEK inhibitors inhibit ERK phosphorylation (P-ERK) and induce BIM levels in *BRAF*-mutant melanoma cell lines. In resistant melanoma cell lines, vemurafenib (BRAF inhibitor) or selumetinib (MEK inhibitor) either fail to suppress P-ERK or resistance emerges through the activity of mammalian target of rapamycin (mTOR), despite P-ERK suppression and BIM induction^9^. This suggests that BIM regulation is MAPK-dependent, but mTOR-independent, and BIM up-regulation is not always sufficient to promote apoptosis^9^. Combining vemurafenib with an mTOR or PI3K inhibitor improved cell killing in *BRAF*-mutant melanomas with ERK-independent resistance to MAPK inhibition^9^. mTOR, a multifunctional 293-kDa serine/threonine protein kinase encoded by the gene *MTOR*, is a downstream effector of PI3K/AKT and promotes cell growth, division, angiogenesis and metabolic reprogramming^9^. The mTOR kinase serves as the catalytic subunit of two multiprotein complexes with distinct functions: mTOR complex 1 (mTORC1), a rapamycin and nutrient-sensitive complex, defined by the regulatory associated protein of mTOR (Raptor), and mTOR complex 2 (mTORC2), a growth-factor-sensitive but nutrient-insensitive complex, defined by the rapamycin-insensitive companion of mTOR (Rictor) (Fig. 1)^10^.

The activity of mTORC1 is regulated by the integration of many signals. For instance, increases in circulating branched-chain amino acids as a result of a high-fat diet, induce mTOR signalling independent of PI3K signalling^11^. In glioblastoma and melanoma cells, diacylglycerol kinase α (DGKα), a lipid kinase converting diacylglycerol to phosphatidic acid, regulates both mTOR activity and *MTOR* mRNA levels via modulation of cyclic adenosine monophosphate (cAMP) (Fig. 1)^12^,^13^. The inhibitory effect of cAMP on mTOR can be also neutralized by phosphodiesterase 4 (PDE4), an enzyme in which two of four isoforms (PDE4A and PDE4D) are increased under hypoxia in lung adenocarcinoma cell lines (Fig. 1)^13^,^14^. Once activated, mTORC1 phosporylates ribosomal S6 kinase 70 kDa (p70S6K) and eIF4E-binding protein 1 (4EBP1) to promote cap-dependent translation and cell growth (Fig. 1).

To further understand the clinical implications of mTOR in *EGFR*-mutant patients, we assessed baseline mRNA levels of *MTOR* by quantitative real-time polymerase chain reaction (qRT-PCR) in 57 *EGFR*-mutant erlotinib or chemotherapy treated NSCLC patients from the EURTAC trial from whom tumour tissue was available^8^. Herein we present updated results of the correlation of *BIM* mRNA alone and in combination with *MTOR* with OS, PFS and response in these 57 *EGFR*-mutant NSCLC patients (training cohort). An independent group of 19 *EGFR*-mutant patients treated with EGFR TKIs was included in the study as a validation cohort for which BIM expression and the phosphorylation state of ribosomal protein S6 (P-S6) were additionally determined by immunohistochemistry. Finally, BIM and mTOR expression were determined in our panel of *EGFR*-mutant lung adenocarcinoma cell lines and correlated with the half maximal inhibitory concentration (IC_50_) of gefitinib. We investigated the effect of gefitinib treatment on BIM expression and mTOR expression and activity. DGKa, PDE4A and PDE4D expression were examined in our cell lines and the results correlated with mTOR expression.

## Results

The EURTAC study enrolled 173 patients with *EGFR* mutations who were randomized to receive erlotinib or standard intravenous chemotherapy with cisplatin or carboplatin plus docetaxel or gemcitabine^1^. Pretreatment tumour specimens were available from 57 of these patients for assessment of *MTOR* mRNA expression. Table 1 shows patient characteristics of the 57 patients included in the present subanalysis. The EURTAC was approved by the Institutional Review Board of each participating centre and written informed consent was obtained from all patients. Among the 48 patients whose *MTOR* mRNA was successfully examined, *MTOR* expression was low (<0.91) or intermediate (0.91–1.97) in 30 (62.5%) and high (>1.97) in 18 (37.5%). Among the 54 patients whose *BIM* mRNA was successfully examined, *BIM* expression was low (<1.83) or intermediate (1.83–2.96) in 36 (66.7%) and high (>2.96) in 18 (33.3%). Evaluation of the expression levels of both *MTOR* and *BIM* was possible in 46 patients.

An independent group of 19 *EGFR*-mutant NSCLC patients receiving erlotinib, gefitinib or afatinib from 2009 to 2014 in Spain, Italy and Colombia was included in the study as a validation cohort. Supplementary Table 1 shows patient characteristics of those 19 patients. Material was available for mRNA analysis of *BIM* and *MTOR* for all of them; *BIM* and *MTOR* mRNA expression was successfully examined in all of them. *MTOR* expression was low (<0.91) or intermediate (0.91–1.97) in 15 (83.3%) and high (>1.97) in 3 (16.7%). *BIM* expression was low (<1.83) or intermediate (1.83–2.96) in 12 (63.2%) and high (>2.96) in 7 (36.8%). Material was available for immunohistochemical analysis of BIM and P-S6 for all 19 patients of the validation cohort and was successfully examined in all of them. Although not statistically significant, a trend for a positive correlation was found between *BIM* mRNA and protein expression (Wilcoxon test two-side *P* value = 0.1161) as well as *MTOR* mRNA and P-S6 expression (Wilcoxon test two-side *P* value = 0.4048) (Supplementary Fig. 1a,b).

### Progression-free survival

On December 9^th^ 2013, median PFS for the 57 patients was 9.7 months (95% confidence intervals [CI], 3.0-13.2) in the erlotinib arm and 6.3 months (95% CI, 5.1–8.3) in the chemotherapy arm *P* = 0.0265). Among the 29 patients treated with erlotinib, PFS was significantly longer for those with high *BIM* than for those with low/intermediate *BIM* mRNA expression 18.5 months, 95% CI, 9.7-not reached [NR] versus [vs] 3.6 months, 95% CI, 1.9–10.4; P = 0.0145) (Fig. 2a). No significant differences in PFS were observed according to *MTOR* mRNA levels. Among the seven erlotinib treated patients with high *BIM* and evaluable *MTOR* expression levels, median PFS was NR (95% CI, 9.7-NR) for those with low/intermediate *MTOR* vs 9.7 months (95% CI, NR) for those with high *MTOR* (*P* = 0.0894). *MTOR* did not affect PFS in patients with low/intermediate *BIM* (Fig. 2b). In the univariate analysis, erlotinib (hazard ratio [HR] = 0.48; 95% CI, 0.25–0.93; *P* = 0.0265) and high *BIM* expression (HR = 0.40; 95% CI, 0.20–0.80; *P* = 0.0095) were associated with longer PFS (Supplementary Table 2). In the multivariate analysis, they both remained markers of longer PFS; HR = 0.49; 95% CI, 0.25–0.96; *P* = 0.0387 and HR = 0.39; 95% CI, 0.19–0.78; *P* = 0.0079 respectively.

With a median follow up of 21.65 (range 3–58) months, median PFS was 13.0 months (95% CI, 8.2–14.9) for the 19 patients of the validation cohort. PFS was significantly longer for those with high *BIM* than for those with low/intermediate *BIM* mRNA expression (15.0 months, 95% CI, 2.6–22.0 vs 9.2 months, 95% CI, 5.4–14.1; *P* = 0.02) (Fig. 2c). No significant differences in PFS were observed according to *MTOR* mRNA expression. Among the 7 patients with high *BIM*, PFS was longer for the four patients with low/intermediate *MTOR* than for the three with high *MTOR* (18.5 months, 95% CI, 14.2.0–53.1 vs 13.0 months, 95% CI, 2.6–15.8; *P* = 0.0939) (Supplementary Fig. 2d).

### Overall survival

On December 9^th^ 2013, with median follow-up of 49.4 months, median OS for the 57 patients was 22.5 months (95% CI, 14.0–30.0) in the erlotinib arm vs 22.1 months (95% CI, 15.4–40.1) in the chemotherapy arm (*P* = 0.4303). OS was significantly longer for the 18 patients with high *BIM* than for the 36 with low/intermediate *BIM* mRNA expression (40.1 months, 95% CI, 14.6–63.0 vs 17.7 months, 95% CI, 13.2–26.8; *P* = 0.010) (Supplementary Fig. 2a). No significant differences in OS were observed according to *MTOR* mRNA levels. Among the 14 patients with high *BIM* and evaluable *MTOR* expression levels, OS was longer for the 11 patients with low/intermediate *MTOR* than for the three with high *MTOR*, though differences were not statistically significant (40.1 months, 95% CI, 8.6-NR vs 20.3 months, 95% CI, 18.1–22.5; P = 0.4848). *MTOR* did not affect OS in patients with low/intermediate *BIM* (Supplementary Fig. 2b). In the univariate analysis for OS, high *BIM* mRNA expression was associated with longer OS (HR = 0.39; 95% CI, 0.19–0.82; *P* = 0.0124), and presence of brain metastases with shorter OS (HR, 2.66; 95% CI 1.10–6.43; *P* = 0.0293) (Supplementary Table 2). In the multivariate analysis, only high *BIM* expression (HR = 0.43; 95% CI, 0.20–0.90; *P* = 0.026) remained a marker of longer OS.

Median OS was 21.6 months (95% CI, 13.2-NR) for the 19 patients of the validation cohort. Though not statistically significant, OS was longer for the 7 patients with high *BIM* than for the 12 with low/intermediate *BIM* mRNA expression (39.2 months, 95% CI, 2.7-NR vs 21.1 months, 95% CI, 12.4-NR; *P* = 0.66). No significant differences in OS were observed according to *MTOR* mRNA levels. Among the seven patients with high BIM mRNA expression, OS was not reached for the four with low/intermediate *MTOR* compared to 15.8 months (95% CI, 13.0–19.0) for the three with high *MTOR* mRNA expression (*P* = 0.0093).

### Response

When the 57 *EGFR*-mutant NSCLC patients of the training cohort were grouped as erlotinib responders and non-responders according to *BIM* mRNA expression, a clear trend emerged: 88.9% of patients with high *BIM* mRNA expression responded to erlotinib vs 22.2% of patients with low/intermediate *BIM* levels (*P* = 0.0027).

When the 19 *EGFR*-mutant NSCLC patients of the validation cohort were grouped as EGFR TKI responders and non-responders according to *BIM* mRNA expression, 85.72% of patients with high BIM responded to EGFR TKIs vs 50.0% patients with low/intermediate BIM (*P* = 0.3240). No differences in response to EGFR TKIs were observed according to *MTOR* mRNA expression in either the training or the validation cohort.

### BIM and mTOR expression and *in vitro* sensitivity to gefitinib

We examined the *in vitro* sensitivity of five EGFR-mutant lung adenocarcinoma cell lines to gefitinib (Table 2). Gefitinib-sensitive PC-9 cells harbour a small in-frame deletion in exon 19 that leads to elimination of an LREA motif in the protein (Del E746–A750). Gefitinib-sensitive H3255 and 11–18 cells harbour a point mutation in exon 21 that substitutes an arginine for leucine at position 858 in the protein (L858R). Gefitinib-insensitive H1975 and H1650 cells, although harbouring the same kinase domain mutations (L858R and Del E746–A750), have additional changes such as T790M (H1975) or phosphatase and tensin homologue (PTEN) loss (H1650).

By matching cell line sensitivity to BIM and mTOR expression, we observed that inhibition concentration of 50% cell viability (IC_50_) induced by gefitinib was increased as mTOR expression increased, in the three sensitive and high BIM expressing *EGFR*-mutant lung adenocarcinoma cell lines, H3255, PC-9 and 11–18. In fact, H3255 cells with high BIM and low mTOR expression (both protein and mRNA) are hypersensitive to gefitinib, yielding IC_50_ values at 10-fold lower concentrations compared to PC-9 and at 100-fold lower concentrations compared to 11–18 (Fig. 3).

To test the ability of gefitinib to induce BIM and inhibit mTOR expression in *EGFR*-mutant cells, we treated cells with gefitinib and performed western blotting and qRT-PCR. Treatment of PC-9 and H3255 cells with gefitinib increased BIM protein expression even at a concentration of 5nM. However changes in mTOR expression levels were not observed in these cells (Fig. 4a). Treatment of PC-9 cells with gefitinib increased *BIM* mRNA expression in a dose- and time-dependent manner but *MTOR* expression was not affected (Fig. 4b). In contrast, gefitinib changed neither BIM nor mTOR expression in the less gefitinib-sensitive 11–18 cells as well as the gefitinib-resistant H1975 and H1650 cells (Fig. 4c). Furthermore in PC-9 and H3255 cells, gefitinib treatment inhibited the phosphorylation of mTOR and p70S6K, while phosphorylation levels of mTOR and p70S6K could not be inhibited below basal levels in 11–18, H1975 and H1650 cells (Fig. 5).

In an exploratory analysis, the protein and mRNA expression levels of DGKa, PDE4A and PDE4D were examined in the five *EGFR*-mutant lung adenocarcinoma cell lines in an effort to explore whether DGKa regulates *MTOR* transcription through modulation of cAMP levels. We also wished to elucidate the role of PDE4 as the predominant cAMP-degrading enzyme. Immunoblotting confirmed that the protein levels of PDE4D and mTOR are similarly increased in 11–18, H1975 and H1650 cells (Supplementary Fig. S3a). By qRT-PCR, *MTOR* mRNA expression showed significant positive correlation with *PDE4D* mRNA expression, with a Pearson correlation coefficient of r = 0.92; *P* = 0.0244 (Supplementary Fig. S3b).

## Discussion

Although expression and degradation of *BIM* are regulated mainly by the MAPK pathway, a variety of other mechanisms can also regulate BIM function, including transcriptional and posttranscriptional regulation to posttranslational modification and epigenetic silencing^3^. For instance, an inverse relationship has been reported between miR-494 and *BIM* expression^15^. AKT may also phosphorylate and suppress the BIM transcription factor FOXO3^3^,^16^. Our findings highlight that pre-treatment assessment of *BIM* levels is able to identify *EGFR*-mutant patients who will benefit more from EGFR TKI treatment.

An additional aim of our study was identification of MAPK-independent mechanisms that may not affect BIM induction but may still affect efficacy of EGFR TKI monotherapy. Among the 29 patients of the training cohort treated with erlotinib, PFS was 18.5 months for those with high *BIM* compared with 3.6 months for those with low/intermediate *BIM* mRNA expression (*P* = 0.0145). Median PFS was not reached for patients with high *BIM* and low/intermediate *MTOR* compared to 9.7 months for those with both high *BIM* and *MTOR*, though differences were not statistically significant (*P* = 0.0894).

In the validation cohort of 19 patients receiving treatment with erlotinib, gefitinib or afatinib, PFS was 15.0 months for those with high *BIM* compared with 9.2 months for those with low/intermediate *BIM* mRNA expression (*P* = 0.02). Among the 7 patients with high *BIM* and evaluable *MTOR* expression levels, PFS was 18.5 months for the four patients with low/intermediate *MTOR* compared to 13.0 months for the three with high *MTOR*, though differences were not statistically significant (*P* = 0.0939).

Interestingly, when we matched gefitinib sensitivity to BIM and mTOR mRNA and protein expression in *EGFR*-mutant lung adenocarcinoma cell lines, we observed that the IC_50_ values of gefitinib increase as the mTOR levels increase in the three sensitive and high BIM expressing cell lines (PC-9, H3255 and 11–18 in Fig. 4). In cells with high mTOR expression, gefitinib did not induce BIM expression and did not suppress mTOR activity (11–18, H1975 and H1650 in Figs 4c and 5b).

mTOR serves as a key signalling hub that integrates signals from several important upstream pathways, making it a bona fide target for molecular therapy^17^. RAF and MEK inhibitor combination has been found to be less effective in *BRAF*-mutant melanoma tumours with MAPK-independent resistance in which ERK is adequately suppressed but alternatively mTOR is activated as estimated by the phosphorylation of p70S6 kinase 1 (S6K1)^9^. Additionally mTOR activity can predict sensitivity of *PIK3CA*-mutant breast tumours to PI3K p110α inhibitors^18^. *MTOR* mutations have also been described as biomarkers for predicting tumour responses to mTOR inhibitors^19^,^20^.

Only rarely does single-agent therapy for cancer result in durable disease control. Patients with low BIM expression could derive only a meagre benefit from treatment with EGFR TKIs alone but could benefit from synthetic lethality combinations, including small molecules that mimic the BH3 motif. A previous study has demonstrated that gefitinib combined with the BH3 mimetic ABT-737 (an analog of navitoclax) substantially increases apoptosis compared with each agent alone in *EGFR*-mutant H1650 cells with low *BIM* expression^21^. Selective Bcl-xL family inhibitors like venetoclax have improved safety and efficacy profiles, compared to their less selective predecessor, navitoclax^22^. Patients with high *BIM* expression could benefit from EGFR TKIs but analysis of *MTOR* could further improve outcomes by selecting patients with high *MTOR* for combination therapy with EGFR TKIs and mTOR inhibitors. Interestingly, the addition of an mTOR inhibitor to BH3 mimetics reduces the expression of the antiapoptotic protein Mcl-1 and allows high *BIM* levels to “prime” tumour cells for apoptosis^23^.

**Figure 1:**
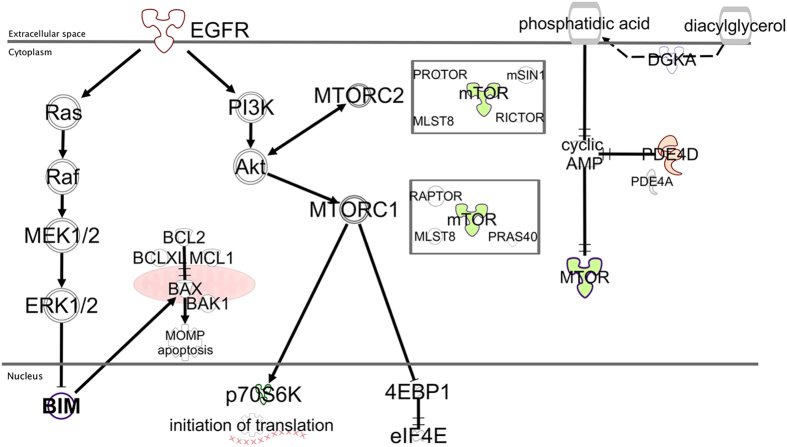
The relationship between the EGFR pathway, apoptosis and the DGKα -PDE4-cAMP-mTOR pathway was designed using the Ingenuity Pathway Analysis (IPA) software (https://www.ingenuity.com/). EGFR stimulates intracellular signalling cascades, such as the RAS/RAF/ERK (MAPK) pathway—which induces BIM proteosomal degradation—and the PI3K/AKT/mTOR pathway. mTOR nucleates a rapamycin and nutrient -sensitive multiprotein complex called mTORC1, and a second growth-factor-sensitive but nutrient-insensitive mTOR-containing complex called mTORC2. Besides mTOR, mTORC1 contains Raptor, mLST8 (also known as GβL), and PRAS40 (proline-rich AKT substrate 40 kDa). mTORC2, like mTORC1, also includes the mLST8 protein, but instead of Raptor, mTORC2 contains the Rictor and mammalian stress-activated protein kinase [SAPK]-interacting (mSIN1) proteins. mTORC2 also contains Protor (protein observed with RICTOR). Ribosomal S6 kinase 70kDa (p70S6K) and eIF4E-binding protein 1 (4EBP1)—both regulators of mRNA translation—are the only extensively described mTORC1 substrates. Phosphorylation of the translational repressor 4EBP1 results in its dissociation from the eukaryotic initiation factor 4E (eIF4E), thereby allowing eIF4E to assemble with other translation initiation factors and initiate cap-dependent translation. mTORC2 directly phosphorylates and activates AKT. BIM activates BAK and BAX, causing activation and mitochondrial outer membrane permeabilization (MOMP). Anti-apoptotic BCL-2 proteins prevent MOMP by binding BIM and other BH3-only proteins as well as activated BAX or BAK. Following MOMP, release of various proteins from the mitochondrial intermembrane space promotes caspase activation and apoptosis. DGKα is a lipid kinase that phosphorylates the lipid diacylglycerol (DAG), transforming it into phosphatidic acid. Phosphatidic acid activates mTOR signalling via a unique pathway involving cAMP. The cAMP-degrading PDE4 enzymes also activate mTOR signalling. mTORC1 promotes survival through translational control of Mcl-1.

**Figure 2:**
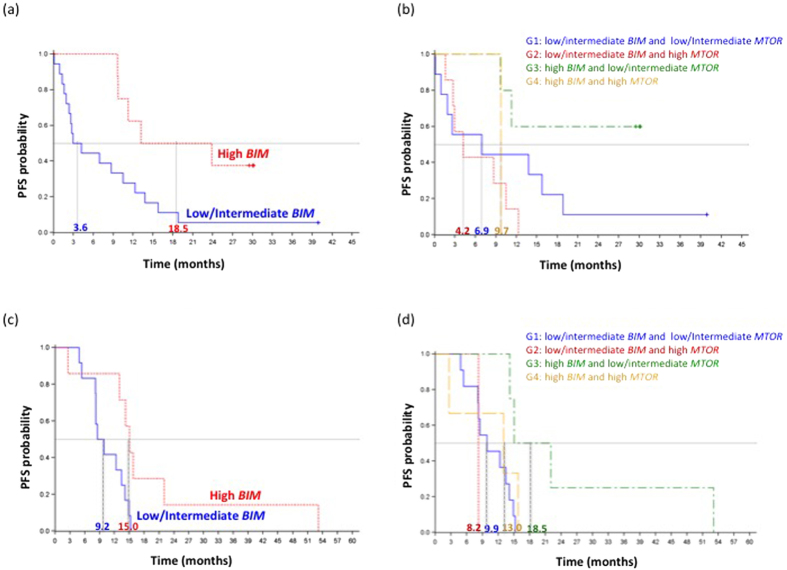
Progression-free survival by *BIM* and *MTOR* mRNA expression levels in the training and validation cohort of patients (**a**).Progression free survival to erlotinib according to *BIM* mRNA levels for the 27 erlotinib treated patients of the training cohort. Median PFS was 18.5 months (95%CI 9.7-NR) for the nine patients with high *BIM* (red line) and 3.6 months (95%CI 1.9–10.4) for the 18 patients with low *BIM* mRNA expression (blue line); *P* = 0.0145; (**b**) Progression-free survival by *BIM* and *MTOR* mRNA levels in 23 *EGFR*-mutant erlotinib-treated NSCLC patients of the training cohort whose *BIM* and *MTOR* mRNA could be evaluated. Median PFS was 6.9 months (95%CI 0.1–18.8) for the nine patients (G1) with low/intermediate *BIM* and *MTOR* and 4.2 months (95%CI 1.6-10.4) for seven patients (G2) with low/intermediate *BIM* and high *MTOR.* Median PFS was NR (95%CI 9.7-NR), for five patients (G3) with high *BIM* and low/intermediate *MTOR* and 9.7 months (95%CI NR) for the only two patients (G4) with high *BIM* and *MTOR.*; *P* = 0.0894. (**c**) Progression free survival to EGFR TKIs according to *BIM* mRNA levels in the 19 *EGFR*-mutant patients of the validation cohort. Median PFS was 15.0 months, (95% CI, 2.6–22.0) in the seven patients with high *BIM* (red line) and 9.2 months, (95% CI, 5.4-14.1) for the 12 patients with low *BIM* mRNA expression (blue line); *P* = 0.02. (**d**) Progression-free survival by *BIM* and *MTOR* mRNA levels in 19 *EGFR*-mutant NSCLC patients of the validation cohort, treated with EGFR TKIs, whose *BIM* and *MTOR* mRNA could be evaluated. Median PFS was 9.9 months (95%CI 5.4–14.1) for the 11 patients (G1) with low/intermediate *BIM* and *MTOR* and 8.2 months (95%CI NR) for one patient (G2) with low/intermediate *BIM* and high *MTOR.* Median PFS was 18.5 months (95%CI 14.2–53.1), for four patients (G3) with high *BIM* and low/intermediate *MTOR* and 13.0 months (95%CI 2.6–15.8) for the three patients (G4) with high *BIM* and *MTOR*; *P* = 0.0939. Note: *BIM* expression levels were divided into high (>2.96), low (<1.83) or intermediate (1.83–2.96). *MTOR* expression levels were divided into high (>1.97), low (<0.91) or intermediate (0.91–1.97).

**Figure 3:**
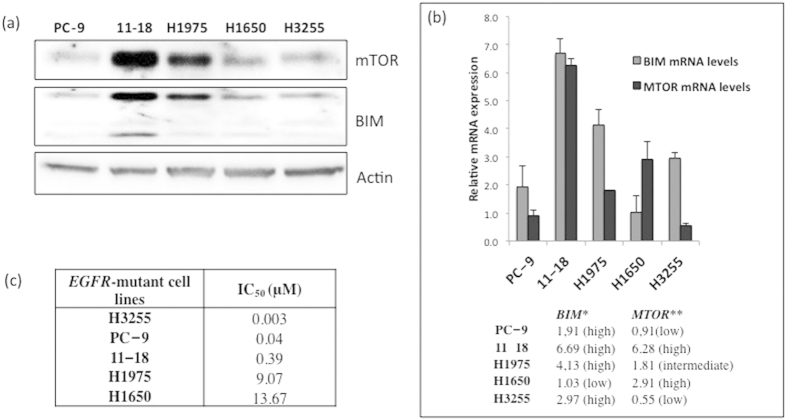
The IC_50_ values for gefitinib in *EGFR*-mutant lung adenocarcinoma cell lines are associated with basal BIM and mTOR expression (protein or mRNA). (**a**) mTOR and BIM expression in *EGFR*-mutant lung adenocarcinoma cell lines. Lysates were prepared and run on gels for western blot with specific antibodies. Actin was used as the loading control. Among the three sensitive *EGFR*-mutant lung adenocarcinoma cell lines, H3255, PC-9 and 11–18, 11–18 is the one with the highest mTOR and BIM protein expression. (**b**) *MTOR* and *BIM* mRNA expression in *EGFR*-mutant lung adenocarcinoma cell lines by qRT-PCR normalized to β-actin. Among the three sensitive *EGFR*-mutant lung adenocarcinoma cell lines, H3255, PC-9 and 11–18, 11–18 has the highest *MTOR* and *BIM* mRNA expression. PC-9, 11–18 and H1975 cells have high *BIM* mRNA expression. H1650 cells have low *BIM* mRNA expression. The two gefitinib resistant *EGFR*-mutant lung adenocarcinoma cell lines, H1975 and H1650, have intermediate and high *MTOR* mRNA expression levels, respectively. Values are the mean ± standard deviation of triplicate experiments. *BIM low, <1.83; *BIM* intermediate, 1.83-2.96; *BIM* high, >2.96; *MTOR* low, <0.91; **MTOR intermediate, 0.91-1.97; and *MTOR* high, >1.97. Error bars indicate the standard deviation. (c). The IC_50_ values for gefitinib increase in the three sensitive *EGFR*-mutant lung adenocarcinoma cell lines, H3255, PC-9 and 11–18, as mTOR expression increases (protein or mRNA). 11–18 are sensitive cells with the highest mTOR expression and IC_50_ value for gefitinib 0.39 μM, a concentration more than 100-fold higher compared to H3255 cells that have the lowest mTOR expression and are hypersensitive to gefitinib (IC_50_ 0.003 μM).

**Figure 4:**
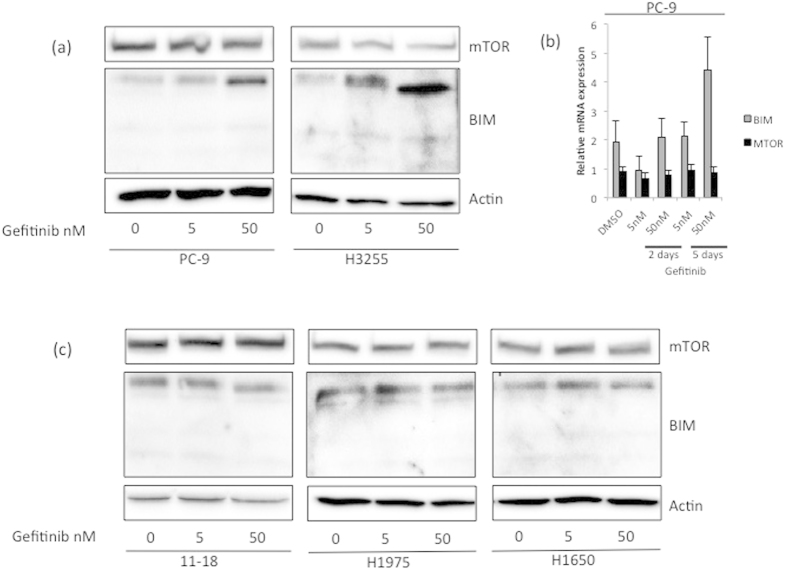
Effect of gefitinib on BIM and mTOR expression. **(a)**. PC-9 and H3255 cells were treated with DMSO vehicle and 5 nM or 50 nM of gefitinib for 24 hours. Lysates were prepared and run on gels for western blot with specific antibodies. Actin was used as the loading control. Incubation of cells with gefitinib induced a dose-dependent increase of BIM but did not change mTOR expression. (**b**) PC-9 cells were treated with indicated concentrations of gefitinib for 5 days. *BIM* and *MTOR* mRNA levels were assessed by qRT-PCR. Incubation of PC-9 cells with gefitinib induced a dose-and time-dependent increase of *BIM* but did not affect *MTOR* mRNA expression. (**c**) 11–18. H1975 and H1650 cells were treated with DMSO vehicle and 5 nM or 50 nM of gefitinib for 24 hours. Gefitinib did not induce BIM or inhibit mTOR expression in the gefitinib-sensitive 11–18 and the gefitinib-resistant H1975 and H1650 cells.

**Figure 5:**
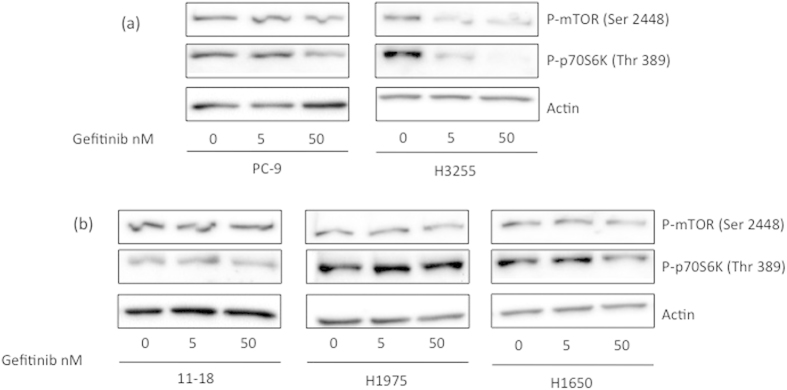
Effect of gefitinib treatment on mTOR signalling. Cells were treated with DMSO vehicle and 5 nM or 50 nM of gefitinib for 24 hours. Lysates were prepared and run on gels for Western blot with phosphorylation-specific antibodies. Actin was used as the loading control. (**a**) Effect of gefitinib on P-mTOR and P-p70S6K in PC-9 and H3255 sensitive cells. Inactivation of P-mTOR on the Ser^2448^ site and of P-p70S6K on the Thr^389^ was observed. (**b**) Effect of gefitinib on P-mTOR and P-p70S6K in the less gefitinib-sensitive 11–18 and the gefitinib-resistant H1975 and H1650 cells. Maintenance of P-mTOR on the Ser^2448^ site and of P-p70S6K on the Thr^389^ was observed after gefitinib treatment in 11–18 and H1975 cells, while suppression of phosphorylation was achieved with the 50nM of gefitinib in H1650 cells.

A better understanding of the DGKα -PDE4-cAMP-mTOR pathway can indicate novel approaches to mTOR inhibition using DGKα or PDE4 inhibitors (Fig. 1)^3^,^12^,^13^,^14^. In the present study, in an exploratory *in vitro* analysis *MTOR* mRNA expression showed significant positive correlation with the *PDE4D* mRNA expression. By immunoblotting, mTOR expression was mainly related with PDE4D expression. Currently the effects of PDE4D in cancer are not fully understood and few studies have examined the role of PDE4D and its inhibitors in cancer therapy. A study revealed that hypoxia via hypoxia-inducible factor 1α regulates PDE4D in lung cancer cell lines, including H1975, and treatment with the first-generation PDE4D inhibitor rolipram decreased cell proliferation^14^. Roflumilast is an oral PDE4 inhibitor used for patients with chronic obstructive pulmonary disease^24^.

The limitations of our study are its retrospective nature and small sample size which limit statistical power. However, the data presented herein provide important biological insights and may be used to refine the predictive role of BIM for outcomes to EGFR TKIs. Pretreatment levels of BIM and mTOR can lead to adding mTOR or PDE4 inhibitors to EGFR TKIs^3^. Also, it is tempting to speculate that PDE4 could be a theranostic marker that warrants further research.

## Methods

The Methods were carried out in accordance with the guidelines defined in the EURTAC study, which was approved by the Institutional Review Board of each participating centre. Written informed consent was obtained from all patients.

### Gene expression analyses

All analyses were carried out centrally at the ISO 15189-certified Pangaea Biotech oncology laboratory located in the Quirón Dexeus University Hospital (Barcelona, Spain). Gene expression analysis of *MTOR* was performed on RNA isolated from the tumour tissue specimens and cell lines. Gene expression analysis of *DGKA, PDE4A* and *PDE4D* was performed on RNA isolated from the cell lines. RNA extraction, retrotranscription analysis, and RT-PCR were performed as previously described and gene expression was examined by quantitative PCR using *β-actin* as housekeeping gene^25^. *BIM* mRNA was previously assessed^8^ and *BIM* mRNA levels were available for 54 of 57 patients in the present analysis. *MTOR* mRNA assessment was possible in 48 patients. From the 50 patients of the validation cohort, 41 of them had sufficient material for mRNA expression. *BIM* and *MTOR* mRNA assessment was possible in 30 and 33 patients respectively.

Primers and probe for gene expression analysis of *β-actin*, *MTOR, DGKA, PDE4A* and *PDE4D* were designed according to their Ref Seq in http://www.ncbi.nlm.nih.gov/sites/entrez?db=gene (Supplementary Table 3). Gene expression of *BIM* was analysed with Hs00708019_s1 (Applied Biosystems).

### Immunohistochemistry

Immunohistochemistry of the tumour samples was performed on 3 μm sections using an automated immunostainer (Ventana BenchMark ULTRA, Ventana Medical Systems). The settings included pretreatment with cell conditioner 1 (CC1) buffer for 76 min, incubation with a BIM antibody (clone Y36, Abcam, ab32158; dilution 1:100) for 40 min, and pretreatment with CC1 buffer for 36 min, incubation with a P-S6 antibody (clone D68F8, Cell Signaling #5364; dilution 1:2000) for 20 min. The detection was performed with DAB detection kit (Ventana Medical Systems) according to manufacturer instruction. Slides were counterstained with hematoxylin and mounted. BIM staining was considered positive when either strong (3+) or moderate (+2) cytoplasmic staining was observed. P-S6 staining was considered positive when only strong (3+) cytoplasmic staining was observed. In addition, protein expression was quantified using the histoscore (HS) method. Briefly, each tumour specimen was scored on a semiquantitative scale ranging from 0 to 300, with the final score resulting from the percentage of tumour cells staining positively (range 0–100) multiplied by staining intensity graded as negative, weak, moderate or strong (range 0–3). The median HS value was used as a cutoff level to discriminate high vs low expression of each biomarker.

### Cell lines

H3255 and 11–18 human lung tumour cell lines were kindly provided by Dr. Daniel Costa (Department of Medicine, Harvard Medical School, Boston, MA) and Dr. Mayumi Ono (Kyushu University, Fukuoka, Japan), respectively. PC-9 human lung tumour cell line was kindly provided by F. Hoffmann-La Roche Ltd. with the authorization of Dr. Mayumi Ono. H1975 and H1650 human lung tumour cell lines were obtained from the American Type Culture Collection (ATCC) collection. Gefitinib was obtained from Selleckchem (USA). A 100 mM stock solution in Dimethylsulfoxide (DMSO) was prepared and stored at −20 °C. All tissue culture materials were obtained from Biological Industries (Kibbutz Beit Haemek, Israel) or Invitrogen (Paisley, Scotland, United Kingdom).

All cell lines were maintained in RPMI medium supplemented with 10% FBS, 50 μg/mL penicillin-streptomycin and 2 mM L-Glutamine. All cells were grown in a humidified atmosphere with 5% CO_2_ at 37 °C. EGFR exons 19 and 21 of all cell lines were sequenced to confirm their status. Cell viability was assessed by the Thiazolyl Blue Tetrazolium Bromide (MTT) (Sigma, St Louis, MO) assay. Cells from each cell line were seeded at 2000 to 6000 per well in 96-well plates. The concentration of gefitinib required for IC_50_ after a 72 h treatment was assessed. After treatment, cells were incubated with medium containing MTT (0.75 mg/mL in medium) for 1–2 h at 37 °C. Culture medium with MTT was removed and formazan crystals reabsorbed in 100 μL DMSO (Sigma, St. Louis, MO). Cell viability was determined by measuring absorbance at 590 nm using a microplate reader (BioWhittaker, Walkersville, MD).

### Western Blotting

For Western blot assays, cells were cultured in cell culture flasks and left untreated or treated as indicated in each experiment. Cells were lysed in ice-cold RIPA buffer [20 mM Tris-HCl (pH:7.5), 150 mM NaCl, 1 mM EDTA, 1mM EGTA, 1% NP40, 1% sodium deoxycholate, 2.5 mM sodium pyrophosphate, 1 mM beta-glycerophosphate, 1 mM Na_3_VO_4_, 1 μg/ml leupeptin, 1 mM PMSF)]. After incubating for 20 minutes at 4 °C, the samples were centrifuged, and the supernatant was kept at—80 °C. Protein concentration was determined by bicinchoninic acid protein assay. Equal amounts of protein from each cell lysate (30 μg/lane) were subjected to SDS polyacrylamide gel electrophoresis (SDS/PAGE) and transferred onto polyvinylidene difluoride membranes (Millipore, New Bedford, MA, USA). The membranes were blocked in Tris-buffered saline containing 5% fat free dry milk and then probed with primary antibodies at 4 °C overnight. After washing, the membrane was incubated with horseradish peroxidase-conjugated secondary antibodies for 2 hours at room temperature. Specific proteins were visualized with enhanced chemiluminescence detection reagent according to the manufacturer’s instructions (Pierce Biotechnology, Rockford, IL, USA). The following antibodies used were from Cell Signaling: BIM (catalog no. 2819), total mTOR (catalog no. 2983), phospho-mTOR [ser2448] (catalog no. 5536) and phospho-P70S6 [thr389] (catalog no. 9234). Other antibodies were Actin (Sigma-Aldrich); DGKa (Abcam, ab197249); PDE4A (Abcam, ab125674); PDE4D (Abcam, ab14613).

### Statistical analysis

The primary endpoint of the study was to examine the potential effects of *BIM* and *MTOR* mRNA expression levels on survival. On December 9^th^ 2013, 135 PFS events had occurred and the results reported here are based on data analyses from that cutoff date. For the OS analysis, patients were not censored at crossover, whereas all patients were censored at crossover for the analysis of PFS. PFS and OS were estimated by means of the Kaplan–Meier method and compared with a nonparametric log-rank test. Based on our previous experience^26^,^27^,^28^, in addition to analysing gene expression as a continuous variable, expression levels were divided into three groups according to their tertiles (inter-quartile ranges [Q1–Q3] were used to describe the data) to explore the risk trend of the gene variable and easily identify groups of gene expression with different risk. A multivariate Cox proportional hazard model was applied with treatment and potential risk factors as covariates, obtaining HRs and their 95% CI. Response rates were compared with the χ^2^ test or Fisher exact test, as required. Each analysis was performed with the use of a two-sided 5% significance level and a 95% CI. Association between *BIM* expression levels and response was evaluated using logistic regression analysis. Association between biomarkers was assessed using a Pearson correlation analysis. The correlation between immunohistochemical and RNA expression analysis has been investigated with the non parametric Mann-Whitney Wilcoxon Two-Sample test; significance was defined at the p < 0.05 level. The statistical analyses were performed using SAS version 9.3 and SPSS version 18.0. The EURTAC study is registered with ClinicalTrials.gov, number NCT00446225.

## Additional Information

**How to cite this article**: Karachaliou, N. *et al.* BIM and mTOR expression levels predict outcome to erlotinib in EGFR-mutant non-small-cell lung cancer. *Sci. Rep.* **5**, 17499; doi: 10.1038/srep17499 (2015).

## Supplementary Material

Supplementary Information

## Figures and Tables

**Table 1:** Patient characteristics of the 57 patients included in the present study.

	Erlotinib(N = 29)	Chemotherapy(N = 28)	Total (N = 57)	P Value Test
**Sex** N(%)				
Female	19 (65.5)	21 (75.0)	40 (70.2)	Chi-Square: 0.4340
Male	10 (34.5)	7 (25.0)	17 (29.8)	
**Age** N(%)				
<65 years	15 (51.7)	12 (42.9)	27 (47.4)	Chi-Square: 0.5027
> = 65 years	14 (48.3)	16 (57.1)	30 (52.6)	
**Smoking status** N(%)				
Never smoked	15 (51.7)	19 (67.9)	34 (59.7)	Fisher: 0.2416
Former smoker	12 (41.4)	6 (21.4)	18 (31.6)	
Current smoker	2 (6.9)	3 (10.7)	5 (8.8)	
ECOG PS* N(%)				
0	9 (31.0)	11 (39.3)	20 (35.1)	Fisher: 0.5693
1	15 (51.7)	15 (53.6)	30 (52.6)	
2	5 (17.2)	2 (7.1)	7 (12.3)	
**Histologic Diagnosis** N(%)				
Adenocarcinoma	28 (96.6)	24 (85.7)	52 (91.2)	Fisher: 0.1086
Bronchioalveolar adenocarcinoma	0 (0.0)	1 (3.6)	1 (1.8)	
Large-cell carcinoma	1 (3.5)	0 (0.0)	1 (1.8)	
Other	0 (0.0)	3 (10.7)	3 (5.3)	
**Clinical Stage** N(%)				
IIIB (malignant effusion)	4 (13.8)	1 (3.6)	5 (8.8)	Fisher: 0.3516
IV	24 (82.8)	27 (96.4)	51 (89.5)	
Unknown	1 (3.5)	0 (0.0)	1 (1.8)	
**Bone metastasis** N(%)				
Yes	7 (24.1)	9 (32.1)	16 (28.1)	Chi-Square: 0.5013
No	22 (75.9)	19 (67.9)	41 (71.9)	
**Brain metastasis** N(%)				
Yes	4 (13.8)	4 (14.3)	8 (14.0)	Fisher: 1.0000
No	25 (86.2)	24 (85.7)	49 (86.0)	
**Type of EGFR mutation** N(%)				
del19	18 (62.1)	19 (67.9)	37 (64.9)	Chi-Square: 0.6471
L858R	11 (37.9)	9 (32.1)	20 (35.1)	

^*^ECOG, Eastern Cooperative Oncology Group.

**Table 2:** IC_50_ values for gefitinib as determined by MTT assay in our panel of *EGFR*-mutant cell lines.

Cells	Mutation	IC_50_* (μM)
H3255	L858R	0.003
PC-9	Del E746–A750	0.04
11–18	L858R	0.39
H1975	L858R, T790M	9.07
H1650	Del E746–A750	13.67

^*^IC_50_: inhibition concentration of 50% cell viability.

## References

[b1] Rosell R. *et al.* Erlotinib versus standard chemotherapy as first-line treatment for European patients with advanced EGFR mutation-positive non-small-cell lung cancer (EURTAC): a multicentre, open-label, randomised phase 3 trial. The lancet oncology 13, 239–246, S1470-2045(11)70393-X 10.1016 (2012). 22285168 10.1016/S1470-2045(11)70393-X

[b2] Mok T. S. *et al.* Gefitinib or carboplatin-paclitaxel in pulmonary adenocarcinoma. The New England journal of medicine 361, 947–957, 10.1056/NEJMoa0810699 (2009). 19692680

[b3] Rosell R., Bivona T. G. & Karachaliou N. Genetics and biomarkers in personalisation of lung cancer treatment. Lancet 382, 720–731, 10.1016/S0140-6736(13)61715-8 (2013). 23972815

[b4] Sequist L. V. *et al.* Genotypic and histological evolution of lung cancers acquiring resistance to EGFR inhibitors. Science translational medicine 3, 75ra26, 3/75/75ra26 10.1126/scitranslmed.3002003 (2011). PMC3132801 21430269

[b5] Faber A. C. *et al.* BIM Expression in Treatment-Naive Cancers Predicts Responsiveness to Kinase Inhibitors. Cancer discovery 1, 352–365 (2011). 22145099 10.1158/2159-8290.CD-11-0106 PMC3229203

[b6] Letai A. *et al.* Distinct BH3 domains either sensitize or activate mitochondrial apoptosis, serving as prototype cancer therapeutics. Cancer cell 2, 183–192 (2002). 12242151 10.1016/s1535-6108(02)00127-7

[b7] Costa D. B. *et al.* BIM mediates EGFR tyrosine kinase inhibitor-induced apoptosis in lung cancers with oncogenic EGFR mutations. PLoS medicine 4, 1669–1679; discussion 1680 (2007). 17973572 10.1371/journal.pmed.0040315 PMC2043012

[b8] Costa C. *et al.* The Impact of EGFR T790M Mutations and BIM mRNA Expression on Outcome in Patients with EGFR-Mutant NSCLC Treated with Erlotinib or Chemotherapy in the Randomized Phase III EURTAC Trial. Clinical cancer research : an official journal of the American Association for Cancer Research 20, 2001–2010, 10.1158/1078-0432.CCR-13-2233 (2014). 24493829

[b9] Corcoran R. B. *et al.* TORC1 suppression predicts responsiveness to RAF and MEK inhibition in BRAF-mutant melanoma. Science translational medicine 5, 196ra198, 10.1126/scitranslmed.3005753 (2013). PMC3867020 23903755

[b10] Laplante M. & Sabatini D. M. mTOR signaling in growth control and disease. Cell 149, 274–293, 10.1016/j.cell.2012.03.017 (2012). 22500797 PMC3331679

[b11] Thomas H. E. *et al.* mTOR inhibitors synergize on regression, reversal of gene expression, and autophagy in hepatocellular carcinoma. Science translational medicine 4, 139ra184, 10.1126/scitranslmed.3003923 (2012). PMC3703151 22539746

[b12] Dominguez C. L. *et al.* Diacylglycerol Kinase alpha Is a Critical Signaling Node and Novel Therapeutic Target in Glioblastoma and Other Cancers. Cancer discovery 3, 782–797, 10.1158/2159-8290.CD-12-0215 (2013). 23558954 PMC3710531

[b13] Rosell R. & Karachaliou N. Lung cancer: Maintenance therapy and precision medicine in NSCLC. Nature reviews. Clinical oncology 10, 549–550, 10.1038/nrclinonc.2013.152 (2013). 23959270

[b14] Pullamsetti S. S. *et al.* Phosphodiesterase-4 promotes proliferation and angiogenesis of lung cancer by crosstalk with HIF. Oncogene 32, 1121–1134, 10.1038/onc.2012.136 (2013). 22525277

[b15] Romano G. *et al.* MiR-494 is regulated by ERK1/2 and modulates TRAIL-induced apoptosis in non-small-cell lung cancer through BIM down-regulation. Proceedings of the National Academy of Sciences of the United States of America 109, 16570–16575, 10.1073/pnas.1207917109 (2012). 23012423 PMC3478630

[b16] Yang J. Y. *et al.* Activation of FOXO3a is sufficient to reverse mitogen-activated protein/extracellular signal-regulated kinase kinase inhibitor chemoresistance in human cancer. Cancer research 70, 4709–4718, 10.1158/0008-5472.CAN-09-4524 (2010). 20484037 PMC2895805

[b17] Marhold M. *et al.* HIF1alpha Regulates mTOR Signaling and Viability of Prostate Cancer Stem Cells. Molecular cancer research : MCR 13, 556–564, 10.1158/1541-7786.MCR-14-0153-T (2015). 25349289

[b18] Elkabets M. *et al.* mTORC1 inhibition is required for sensitivity to PI3K p110alpha inhibitors in PIK3CA-mutant breast cancer. Science translational medicine 5, 196ra199, 10.1126/scitranslmed.3005747 (2013). PMC3935768 23903756

[b19] Grabiner B. C. *et al.* A diverse array of cancer-associated MTOR mutations are hyperactivating and can predict rapamycin sensitivity. Cancer discovery 4, 554–563, 10.1158/2159-8290.CD-13-0929 (2014). 24631838 PMC4012430

[b20] Wagle N. *et al.* Activating mTOR mutations in a patient with an extraordinary response on a phase I trial of everolimus and pazopanib. Cancer discovery 4, 546–553, 10.1158/2159-8290.CD-13-0353 (2014). 24625776 PMC4122326

[b21] Cragg M. S., Kuroda J., Puthalakath H., Huang D. C. & Strasser A. Gefitinib-induced killing of NSCLC cell lines expressing mutant EGFR requires BIM and can be enhanced by BH3 mimetics. PLoS medicine 4, 1681–1689; discussion 1690 (2007). 17973573 10.1371/journal.pmed.0040316 PMC2043013

[b22] Leverson J. D. *et al.* Exploiting selective BCL-2 family inhibitors to dissect cell survival dependencies and define improved strategies for cancer therapy. Science translational medicine 7, 279ra240, 10.1126/scitranslmed.aaa4642 (2015). 25787766

[b23] Faber A. C. *et al.* Assessment of ABT-263 activity across a cancer cell line collection leads to a potent combination therapy for small-cell lung cancer. Proceedings of the National Academy of Sciences of the United States of America 112, E1288–1296, 10.1073/pnas.1411848112 (2015). 25737542 PMC4371986

[b24] Martinez F. J. *et al.* Effect of roflumilast on exacerbations in patients with severe chronic obstructive pulmonary disease uncontrolled by combination therapy (REACT): a multicentre randomised controlled trial. Lancet, 10.1016/S0140-6736(14)62410-7 (2015). 25684586

[b25] Rosell R. *et al.* Pretreatment EGFR T790M Mutation and BRCA1 mRNA Expression in Erlotinib-Treated Advanced Non-Small-Cell Lung Cancer Patients with EGFR Mutations. Clinical cancer research : an official journal of the American Association for Cancer Research 17, 1160–1168, 1078-0432.CCR-10-2158 10.1158 (2011). 21233402 10.1158/1078-0432.CCR-10-2158

[b26] Boukovinas I. *et al.* Tumor BRCA1, RRM1 and RRM2 mRNA expression levels and clinical response to first-line gemcitabine plus docetaxel in non-small-cell lung cancer patients. PloS one 3, e3695 (2008). 19002265 10.1371/journal.pone.0003695 PMC2579656

[b27] Margeli M. *et al.* The prognostic value of BRCA1 mRNA expression levels following neoadjuvant chemotherapy in breast cancer. PloS one 5, e9499 (2010). 20209131 10.1371/journal.pone.0009499 PMC2831058

[b28] Rosell R. *et al.* Customized treatment in non-small-cell lung cancer based on EGFR mutations and BRCA1 mRNA expression. PloS one 4, e5133 (2009). 19415121 10.1371/journal.pone.0005133 PMC2673583

