# Author Correction: BIM and mTOR expression levels predict outcome to erlotinib in EGFR-mutant non-small-cell lung cancer

**DOI:** 10.1038/s41598-023-30374-9

**Published:** 2023-03-03

**Authors:** Niki Karachaliou, Jordi Codony-Servat, Cristina Teixidó, Sara Pilotto, Ana Drozdowskyj, Carles Codony-Servat, Ana Giménez-Capitán, Miguel Angel Molina-Vila, Jordi Bertrán-Alamillo, Radj Gervais, Bartomeu Massuti, Teresa Morán, Margarita Majem, Enriqueta Felip, Enric Carcereny, Rosario García-Campelo, Santiago Viteri, María González-Cao, Daniela Morales-Espinosa, Alberto Verlicchi, Elisabetta Crisetti, Imane Chaib, Mariacarmela Santarpia, José Luis Ramírez, Joaquim Bosch-Barrera, Andrés Felipe Cardona, Filippo de Marinis, Guillermo López-Vivanco, José Miguel Sánchez, Alain Vergnenegre, José Javier Sánchez Hernández, Isabella Sperduti, Emilio Bria, Rafael Rosell

**Affiliations:** 1 Instituto Oncológico Dr Rosell, Quiron-Dexeus University Hospital, Barcelona, Spain; 2 Pangaea Biotech, Barcelona, Spain; 3 Department of Medical Oncology, University of Verona, Azienda Ospedaliera Universitaria Integrata, Verona, Italy; 4 Pivotal, Madrid, Spain; 5 Centre François Baclesse, Caen, France; 6 Hospital General de Alicante, Alicante, Spain; 7 Catalan Institute of Oncology, Hospital Germans Trias i Pujol, Badalona, Spain; 8 Hospital de Sant Pau, Barcelona, Spain; 9 Hospital Vall d’Hebron, Barcelona, Spain; 10 Complexo Hospitalario Universitario La Coruña, La Coruña, Spain; 11 Ospedale Santa Maria delle Croci, Ravenna, Italy; 12 Department of Medical and Surgical Sciences, Institute of Respiratory Diseases, University of Foggia, Foggia, Italy; 13 Human Pathology Department, Medical Oncology Unit, University of Messina, Messina, Italy; 14 Catalan Institute of Oncology, Hospital Josep Trueta, Girona, Spain; 15 Clinical and Traslational Oncology Group, Institute of Oncology, Clínica del Country, Bogotá, Colombia; 16 Divisione di Oncologica Toracica, Direttore, Istituto Europeo di Oncologia—IEO, Milano, Italy; 17 Chief, Medical Oncology Service, Hospital de Cruces, Barakaldo, Vizcaya Spain; 18 Medical Oncology Service, Hospital de la Princesa, Madrid, Spain; 19 Hôpital du Cluzeau, Limoges, France; 20 Unidad de Investigación en Salud Pública CIDICS-UANL, Monterrey, Mexico; 21 Biostatistics, Regina Elena National Cancer Institute, Rome, Italy; 22 Molecular Oncology Research (MORe) Foundation, Barcelona, Spain; 23 Germans Trias i Pujol Health Sciences Institute and Hospital, Campus Can Ruti, Barcelona, Spain

Correction to: *Scientific Reports* 10.1038/srep17499, published online 07 December 2015

This Article contains an error in Figure 3A, where the mTOR panel is an inadvertent duplication of the BIM panel.

The corrected Figure 3 and accompanying legend appear below.

**Figure 3:**
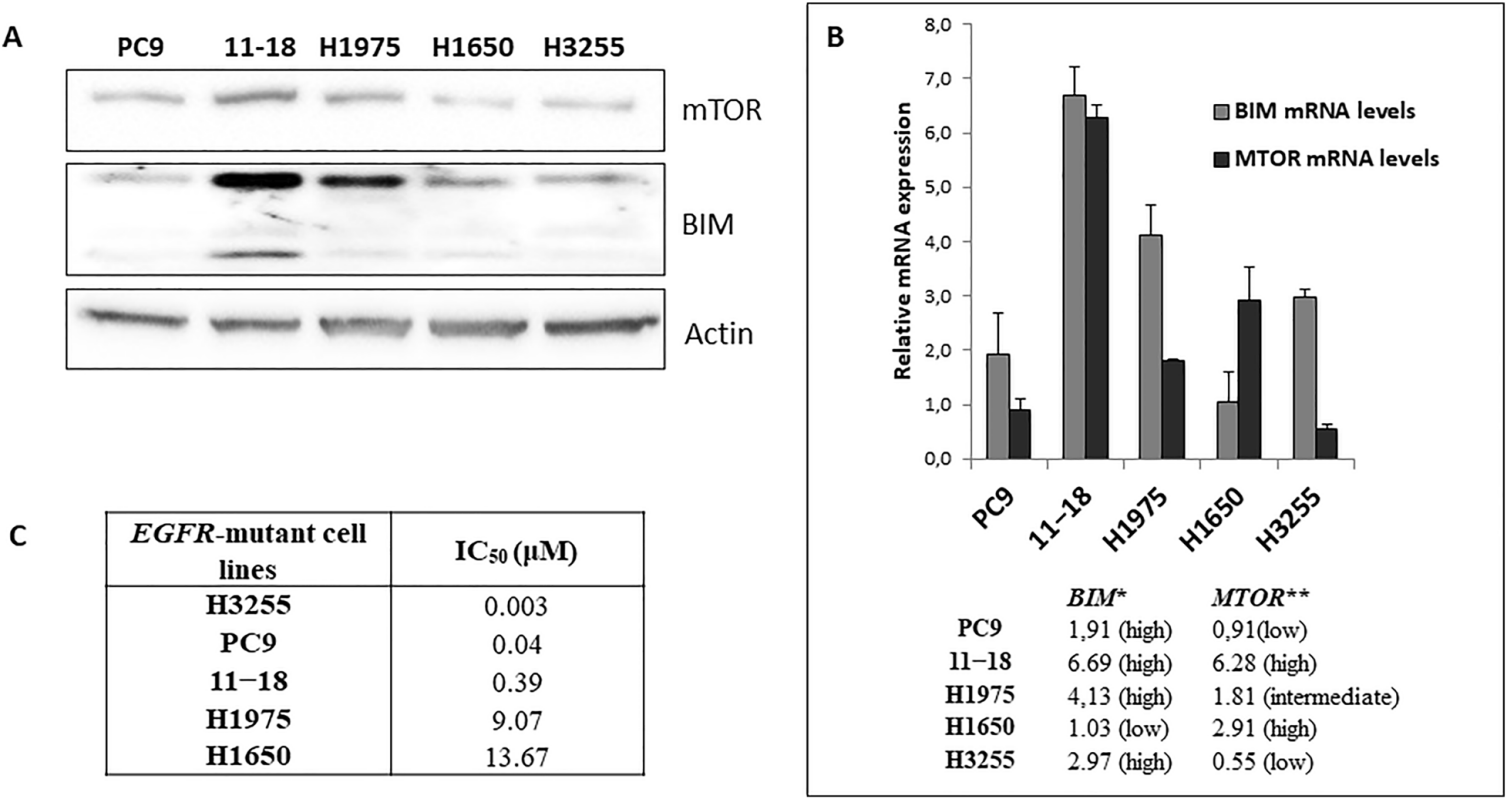
The IC_50_ values for gefitinib in *EGFR*-mutant lung adenocarcinoma cell lines are associated with basal BIM and mTOR expression (protein or mRNA). (**a**) mTOR and BIM expression in *EGFR*-mutant lung adenocarcinoma cell lines. Lysates were prepared and run on gels for western blot with specific antibodies. Actin was used as the loading control. Among the three sensitive *EGFR*-mutant lung adenocarcinoma cell lines, H3255, PC-9 and 11–18, 11–18 is the one with the highest mTOR and BIM protein expression. (**b**) *MTOR* and *BIM* mRNA expression in *EGFR*-mutant lung adenocarcinoma cell lines by qRT-PCR normalized to β-actin. Among the three sensitive *EGFR*-mutant lung adenocarcinoma cell lines, H3255, PC-9 and 11–18, 11–18 has the highest *MTOR* and *BIM* mRNA expression. PC-9, 11–18 and H1975 cells have high *BIM* mRNA expression. H1650 cells have low *BIM* mRNA expression. The two gefitinib resistant *EGFR*-mutant lung adenocarcinoma cell lines, H1975 and H1650, have intermediate and high *MTOR* mRNA expression levels, respectively. Values are the mean ± standard deviation of triplicate experiments. *BIM low, < 1.83; *BIM* intermediate, 1.83–2.96; *BIM* high, > 2.96; *MTOR* low, < 0.91; **MTOR intermediate, 0.91–1.97; and *MTOR* high, > 1.97. Error bars indicate the standard deviation. (**c**) The IC_50_ values for gefitinib increase in the three sensitive *EGFR*-mutant lung adenocarcinoma cell lines, H3255, PC-9 and 11–18, as mTOR expression increases (protein or mRNA). 11–18 are sensitive cells with the highest mTOR expression and IC_50_ value for gefitinib 0.39 μM, a concentration more than 100-fold higher compared to H3255 cells that have the lowest mTOR expression and are hypersensitive to gefitinib (IC_50_ 0.003 μM).

In addition, the Supplementary Information file published with this Article contains an error in Supplementary Figure 3A, where the mTOR panel is inadvertently duplicated from the BIM panel. The corrected Supplementary Information file is provided below.

## Supplementary Information

Supplementary Information.

