# Supplementary material for: Author Correction: BIM and mTOR expression levels predict outcome to erlotinib in EGFR-mutant non-small-cell lung cancer
Source: Sci Rep. 2023 Mar 3;13:3620. doi: 10.1038/s41598-023-30374-9 (PMC9984426; doi:10.1038/s41598-023-30374-9)
Supplement: Supplementary file 1 — Supplementary Information. [file 41598_2023_30374_MOESM1_ESM.pdf]

BIM and mTOR expression levels predict outcome to erlotinib in EGFR-mutant non-small-cell lung cancer

Supplementary Material

Supplementary Table 1. Patient characteristics of the 19 patients included in the validation cohort

**Clinical parameter**	N(%)
**Sex**	
Female	14 (73.7)
Male	5 (26.3)
**Age group**	
<65 years	11 (77.9)
>= 65 years	8 (42.1)
**Smoking status**	
Never smoker	15 (78.9)
Former smoker	3 (15.8)
Current Smoker	1 (5.3)
**ECOG PS**	
0-1	12 (63.2)
2	7 (36.8)
**Histologic Diagnosis**	
Adenocarcinoma	19 (100.0)
**Clinical Stage**	
IIIA	1 (5.3)
IIIB	2 (10.5)
IV	16 (84.2)
**Bone metastasis**	
Yes	9 (47.4)
No	10 (52.6)
**Brain metastasis**	
Yes	7 (36.8)
No	12 (63.2)
**Other metastasis**	
Yes	12 (63.2)
No	7 (36.8)

**Type of EGFR mutation**	
del19	12 (63.2)
L858R	7 (36.8)
**Type of EGFR TKI**	
Erlotinib	13 (68.4)
Gefitinib	5 (26.3)
Afatinib	1 (5.3)
**Response**	
Complete response	2 (10.5)
Partial response	10 (52.6)
Stable disease	7 (36.8)

Supplementary Table 2: Univariate analyses of progression-free and overall survival in 57 patients from the EURTAC trial included in the present study.

	PFS		OS	
Variable	HR (95%CI)	P	HR (95%CI)	P
**Gender**				
Female	1.00	-	1.00	
Male	1.14 (0.60, 2.18)	.6859	1.20 (0.63, 2.28)	.5773
**Smoking history**				
Current smoker	1.00		1.00	
Never smoked	1.10 (0.33, 3.68)	.8737	0.39 (0.15, 1.05)	.1791
Former smoker	1.05 (0.30, 3.68)	.9391	0.46 (0.17, 1.31	.1472
**ECOG performance status**				
1,2	1.00		1.00	
0	0.67 (0.35, 1.27)	.2156	0.78 (0.41, 1.49)	.4554
**Treatment**				
Chemotherapy	1.00		1.00	
Erlotinib	0.48 (0.25, 0.93)	.0265	1.28 (0.69, 2.35)	.4321
**Bone metastasis**				
No	1.00		1.00	
Yes	1.50 (0.77, 2.92)	.2298	1.19 (0.60, 2.38)	.6178
**Brain metastasis**				
No	1.00		1.00	
Yes	2.31 (0.89, 5.97)	.0749	2.66 (1.10, 6.43)	.0293
**Type of EGFR mutation**				
del19	1.00		1.00	
L858R	0.69(0.36, 1.30)	.2467	0.87(0.46, 1.64)	.6659
***BIM* expression**				
Low/intermediate	1.00		1.00	
High	0.40 (0.20, 0.80)	.0095	0.39 (0.19, 0.82)	.0124

Supplementary Table 3. Primers and probes used for gene expression analyses

GENES	REFSEQ		PRIMERS	PROBES
β-actin	NM_001101.3	F	5´ TGAGCGCGGCTACAGCTT 3´	6FAM 5´ACCACCACGGCCGAGCGG 3´ TAMRA
		R	5´ TCCTTAATGTCACGCACGATTT 3´	
MTOR	NM_004958	F	5´ AGGCCGCATTGTCTCTATCAA 3´	6FAM 5´ TGCAATCCAGCTGTTTG 3´ MGB
		R	5´GCAGTAAATGCAGGTAGTCATCCA 3´	
DGKA	NM_001345	F	5´ CCCAGTGATTTTGCCCAGC 3´	6FAM 5´ AATACTCCACCAAAAAG 3´ MGB
		R	5´ CCATCCTCGAAGAGCTTTAGGA3´	
PDE4A	NM_001111307	F	5´ GGAGACCATGCAGACCTATCG 3´	6FAM 5´ TGGCCTCGCACAAGT 3´ MGB
		R	5´ GCTCACGGTTCAACATCCTTTT3´	
PDE4D	NM_ 001104631	F	5´ ATCATCCTGGTGTGTCCAATCA 3´	6FAM 5´ TCTGATCAATACAAACTCT 3´ MGB
		R	5´ GGAATCATTGTACATCAAGGCAAGT3´	

Supplementary Figures:

Supplementary Figure 1:

(a)

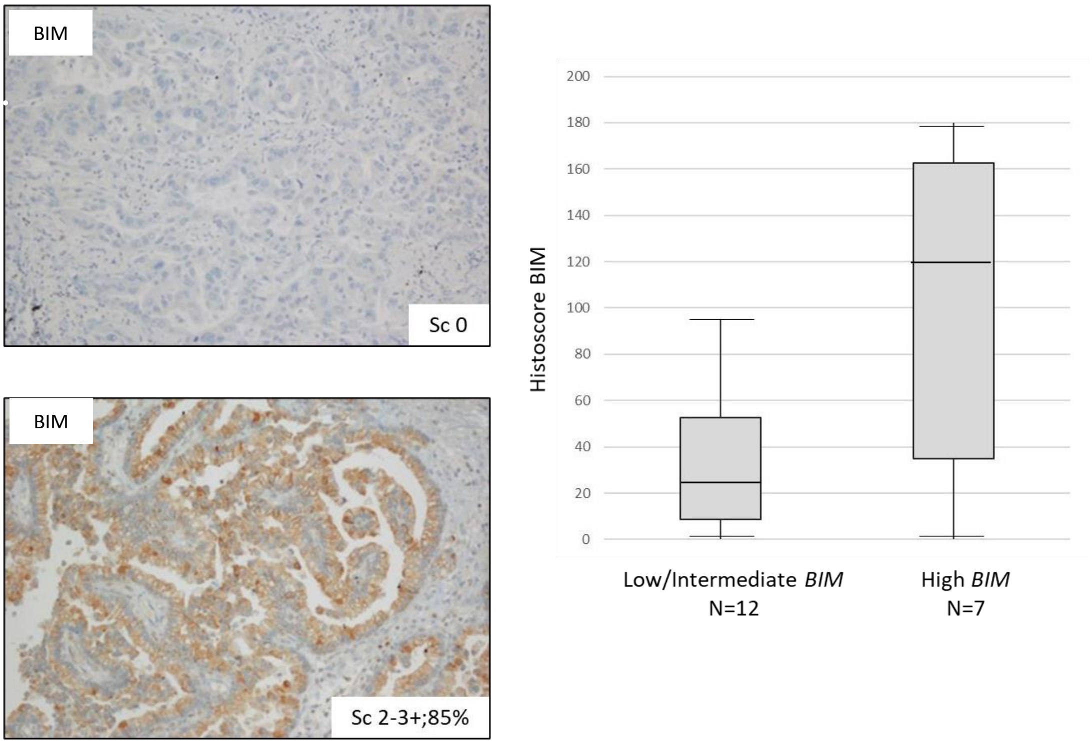

(b)

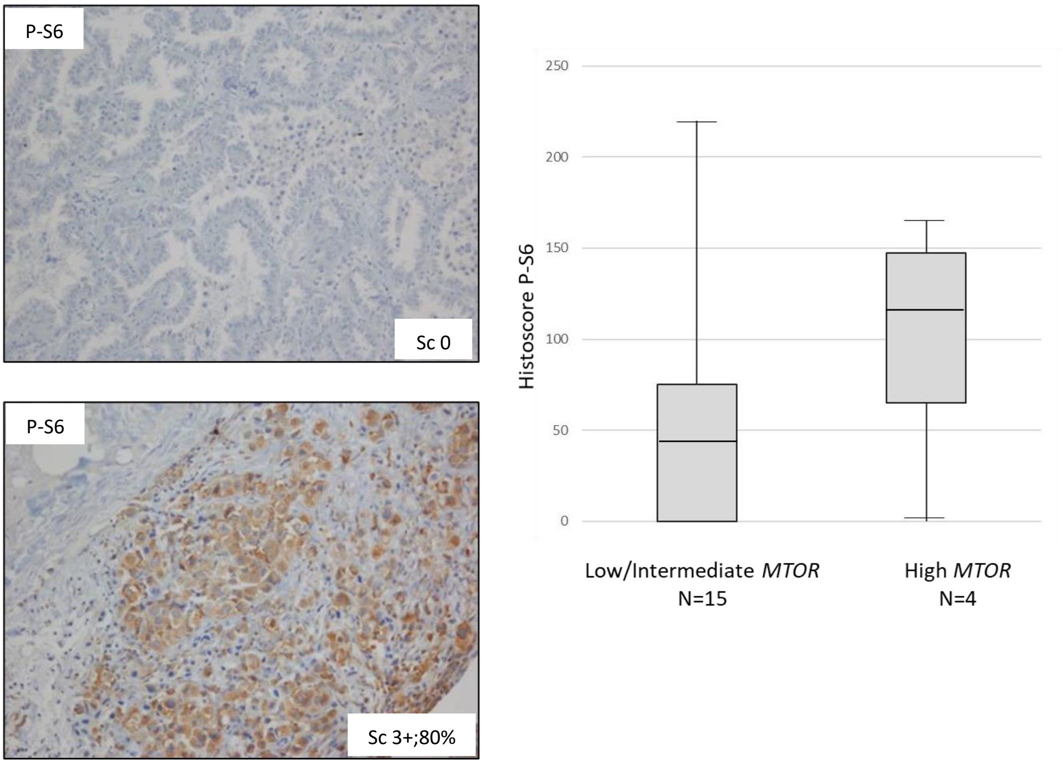

Examples of immunohistochemical analysis and correlation between mRNA and protein expression. (a). Left: Representative cases of BIM protein expression. BIM staining was considered positive when either strong (3+) or moderate (+2) cytoplasmic staining was observed (scale bar 100μm).

Representative IHC staining images for a negative case (score 0; Sc 0) and a positive case (score 2-3+ in 85% of the cells; Sc 2-3+). Right: The correlation between BIM protein and mRNA expression is presented by box- and -whisker plots. Medians, interquartile, maximum and minimum are shown. Though not statistically significant a trend for a positive correlation was found between *BIM* mRNA and protein expression (Wilcoxon test two-side *P* value =.1161). **(b)**. Representative cases of P-S6 protein expression with anti-p-S6 (Ser240/244). P-S6 staining was considered positive when only strong (3+) cytoplasmic staining was observed (scale bar 100μm). Representative IHC staining images for a negative case (score 0; Sc 0) and a positive case (score 3+ in 80% of the cells; Sc 3+). Right: The correlation between P-S6 protein and *MTOR* mRNA expression is presented by box- and -whisker plots. Medians, interquartile, maximum and minimum are shown. Though not statistically significant a trend for a positive correlation was found between *MTOR* mRNA and P-S6 protein expression (Wilcoxon test two-side *P* value = .4048).

Supplementary Figure 2:

a)

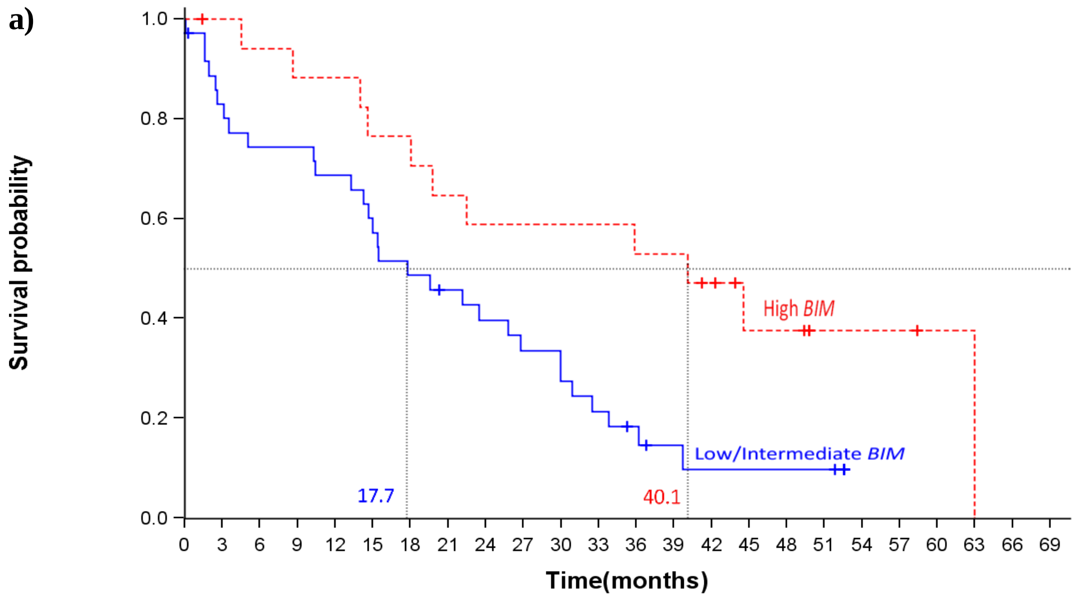

b)

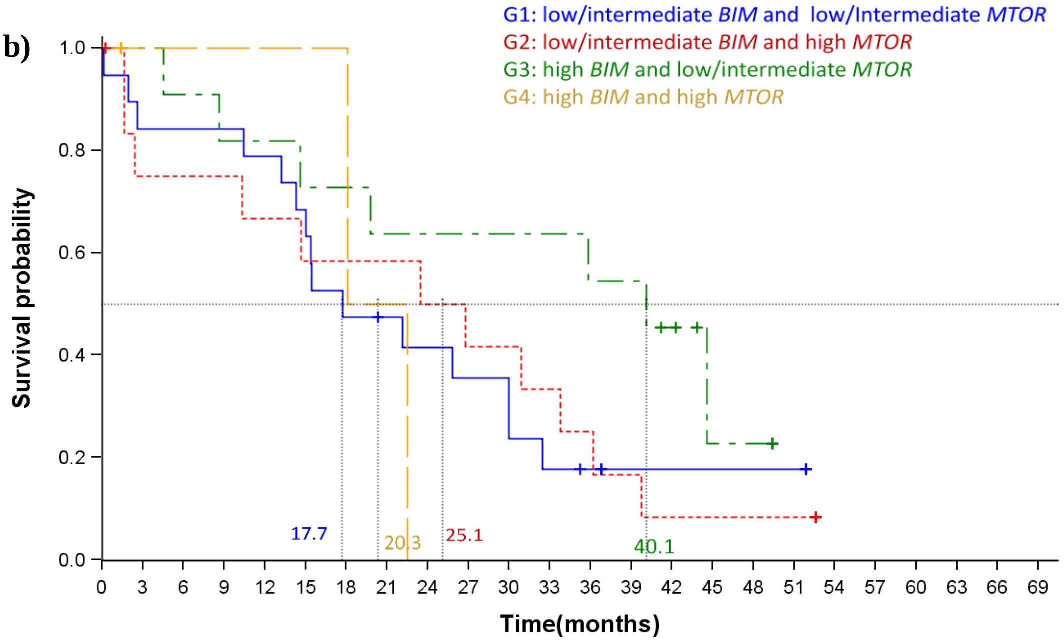

Overall survival, according to *BIM* and *MTOR* mRNA expression levels for the training cohort of patients form the EURTAC study. (a). Overall survival according to *BIM* mRNA levels for all the 57 *EGFR*-mutant NSCLC patients included in the present analysis. OS was 40.1 months (95%CI 14.6-63.0) for the 36 patients with high *BIM* (red line) and 17.7 months (95%CI 13.2-26.8) for the 18 patients with low/intermediate *BIM* mRNA expression (blue line); *P*=.010.

(b). Overall survival by *BIM* and *MTOR* mRNA levels for the 46 *EGFR*-mutant NSCLC patients of the training cohort in whom both *BIM* and *MTOR* mRNA evaluation was possible. Median OS was 17.7 (95%CI 13.3-30), for 19 patients (G1) with low/intermediate both *BIM* and *MTOR* and 25.1 months (95%CI 1.6-36.2) for the 13 patients (G2) with low/intermediate *BIM* and high *MTOR.* Median OS was 40.1 months (95%CI 8.6-NR) for 11 patients (G3) with high *BIM* and low/intermediate *MTOR* and 20.3 months (95%CI 18.1-22.5) for three patients (G4) with high both *BIM* and *MTOR*; *P*=.2497.

Note Supplementary Figure 2: *BIM* expression levels were divided into high (>2.96) and low (<1.83) or intermediate (1.83-2.96). *MTOR* expression levels were divided into high (>1.97) and low (<0.91) or intermediate (0.91-1.97).

Supplementary Figure 3:

(a)

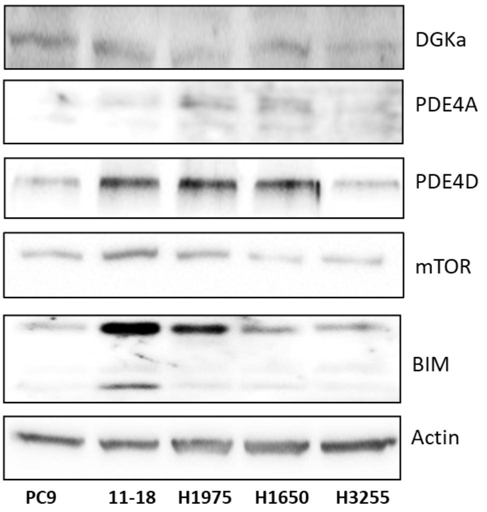

(b)

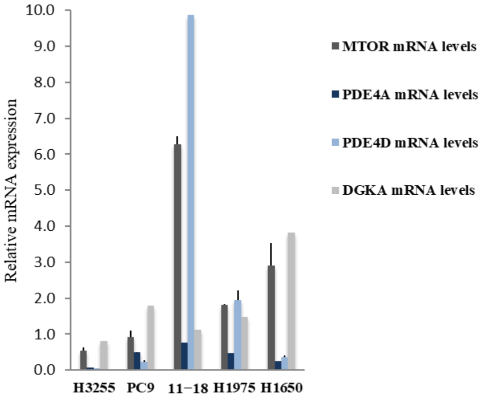

***EGFR*-mutant cell lines**	***MTOR***	***PDE4A***
**H3255**	0.55	0.08
**PC9**	0.91	0.49
**11-18**	6.28	0.77
**H1975**	1.81	0.48
**H1650**	2.91	0.26

***EGFR*-mutant cell lines**	***PDE4D***	***DGKA***
**H3255**	0.06	0.81
**PC9**	0.24	1.78
**11-18**	9.86	1.13
**H1975**	1.95	1.47
**H1650**	0.36	3.82

Correlation of MTOR expression with the expression of DGKA and PDE4. (a). mTOR and DGKa, PDE4A and PDE4D expression in *EGFR*-mutant lung adenocarcinoma cell lines. Cell lysates were collected and probed with anti-mTOR and anti-DGKa, anti-PDE4A anti-PDE4D antibodies. Actin was used as the loading control. The protein levels of PDE4D and mTOR are similarly increased in 11-18, H1975 and H1650 cells. **(b)**. *MTOR*, *DGKA*, *PDE4A and PDE4D* mRNA expression in *EGFR*-mutant lung adenocarcinoma cell lines by qRT-PCR normalized to β-actin. Correlation between the four biomarkers was assessed using Pearson´s correlation analysis. There was a significant positive correlation between *MTOR* and *PDE4D* mRNA expression (r=0.92, *P*=.0244). Pearson correlation coefficients (r) of 0.75 (P=.1418) and 0.05 (P=.09413) were found between *MTOR* and *PDE4A* and *MTOR* and *DGKA*, respectively. Values are the mean± standard deviation of triplicate experiments. Error bars indicate the standard deviation.
